# P-2357. Respiratory Syncytial Virus (RSV) Disease Burden among Adults in Primary Care Settings in High-income Countries: a Systematic Review and Modelling Study

**DOI:** 10.1093/ofid/ofae631.2508

**Published:** 2025-01-29

**Authors:** You Li, Sebastien Kenmoe, Elizabeth Begier, Pia Wahi-Singh, Bhanu Wahi-Singh, Caihua Liang, Reiko Sato, Bradford D Gessner, Harish Nair

**Affiliations:** Nanjing Medical University, Nanjing, Jiangsu, China (People's Republic); University of Edinburgh, Edinburgh, Scotland, United Kingdom; Pfizer Vaccines, Dublin, Dublin, Ireland; University of Edinburgh, Edinburgh, Scotland, United Kingdom; University of Edinburgh, Edinburgh, Scotland, United Kingdom; Pfizer Inc, New York, New York; Pfizer, Inc., Collegeville, Pennsylvania; Pfizer Biopharma Group, Collegeville, Pennsylvania; University of Edinburgh, Edinburgh, Scotland, United Kingdom

## Abstract

**Background:**

Data on RSV disease burden in adults remain scarce and mostly focus on hospitalisation. We aimed to estimate RSV disease burden in primary-care settings among adults in high-income countries.

**Figure 1.**
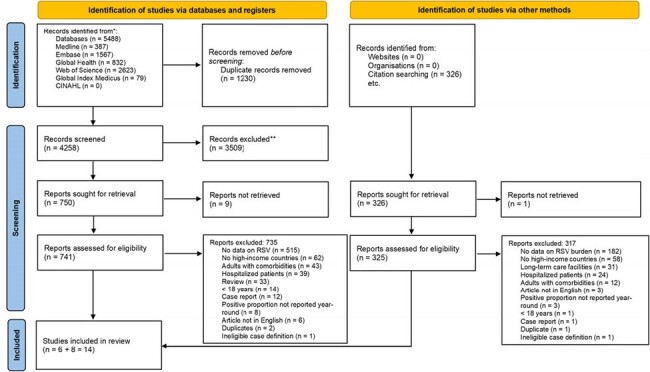
PRISMA flowchart

**Methods:**

We conducted a systematic review to include studies that reported RSV disease burden in primary-care settings; studies of emergency department (ED) visits were also included but analysed separately. We searched MEDLINE, Embase, Global Health, CINAHL, Web of Science, and Global Index Medicus for eligible studies published between 1 Jan 1996 and 30 Nov 2022 and conducted citation searching of identified studies. Using a random-effects meta-analysis, we synthesised RSV incidence rate, positive proportion, and incidence-to-hospitalisation ratio. When estimating RSV incidence rate and positive proportion, we adjusted for RSV under-ascertainment related to clinical specimens and diagnostic tests used in individual studies by a previously published two-stage statistical framework. We used the Joanna Briggs Institute critical appraisal checklist for assessing study quality.

**Figure:**
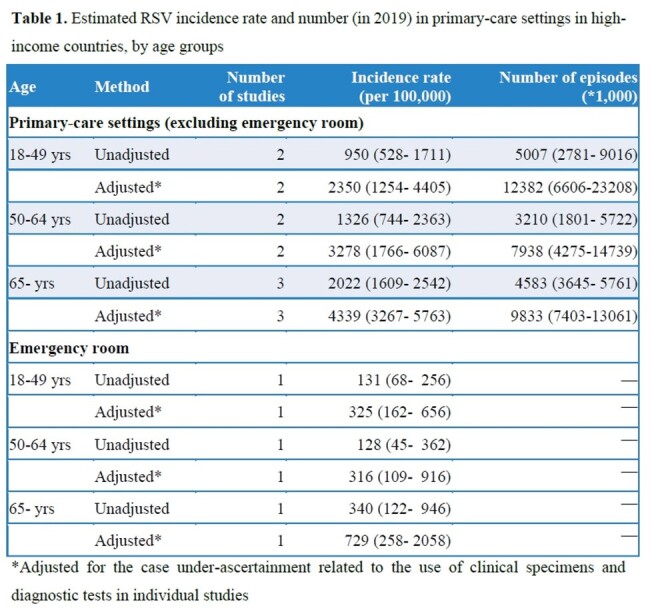

**Results:**

We included 14 studies (Figure 1), with 13 studies rated as high-quality. RSV incidence rate increased with increasing age in primary-care settings and in ED; the adjusted annual incidence rate in primary-care settings ranged from 2,350 (95% CI: 1,254–4,405) per 100,000 in 18–49 year-olds to 4,339 (3,267–5,763) in adults ≥65 years (Table 1). The RSV positive proportion in acute respiratory infection episodes in primary-care settings was higher among those aged ≥65 years than 18–49 years (Table 2). In adults ≥65 years, 6.2% (1.4–23.8) of RSV episodes in primary-care settings led to hospitalisation, equivalent to one patient progressing to hospitalisation for every 16 outpatient visits. According to five studies (total N=413; age: >60 or >65 years), no RSV-associated deaths occurred in primary-care settings.

**Figure:**
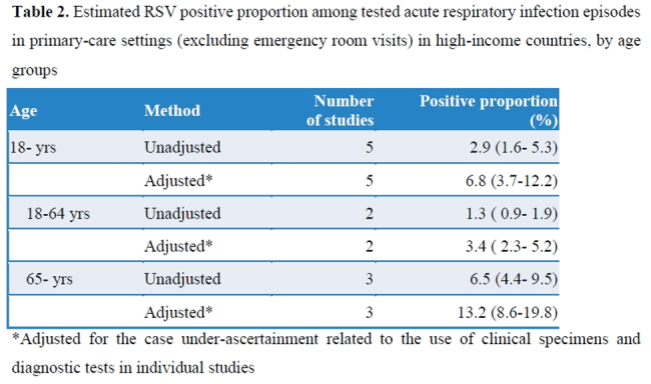

**Conclusion:**

RSV disease burden in primary-care settings is substantial among adults in high-income countries, particularly for older adults. Further larger studies are needed to assess mortality risk following RSV-related outpatient visits. This study’s estimates have important implications for evaluating RSV prevention programmes’ impact.

**Disclosures:**

You Li, Pfizer: Advisor/Consultant Elizabeth Begier, MD, M.P.H., Pfizer Vaccines: Employee|Pfizer Vaccines: Stocks/Bonds (Private Company) Caihua Liang, MD, PhD, Pfizer: Stocks/Bonds (Private Company) Reiko Sato, PhD, Pfizer Inc: employee|Pfizer Inc: Stocks/Bonds (Private Company) Bradford D. Gessner, M.D., M.P.H., Pfizer: Employee|Pfizer: Stocks/Bonds (Public Company) Harish Nair, n/a, Pfizer: Advisor/Consultant

